# Down-regulation of GATA1-dependent erythrocyte-related genes in the spleens of mice exposed to a space travel

**DOI:** 10.1038/s41598-019-44067-9

**Published:** 2019-05-21

**Authors:** Kenta Horie, Hiroki Sasanuma, Takashi Kudo, Shin-ichiro Fujita, Maki Miyauchi, Takahisa Miyao, Takao Seki, Nobuko Akiyama, Yuki Takakura, Miki Shimbo, Hyojung Jeon, Masaki Shirakawa, Dai Shiba, Nobuaki Yoshida, Masafumi Muratani, Satoru Takahashi, Taishin Akiyama

**Affiliations:** 1 RIKEN Center for Integrative Medical Sciences, Yokohama, 230-0045 Japan; 2 Laboratory of Developmental Genetics, Institute of Medical Science, The University of Tokyo, Tokyo, 108-8639 Japan; 3 Mouse Epigenetics Project, ISS/Kibo experiment, Japan Aerospace Exploration Agency (JAXA), Ibaraki, 305-8505 Japan; 4 Laboratory Animal Resource Center and Department of Anatomy and Embryology, Faculty of Medicine, University of Tsukuba, Ibaraki, 305-8575 Japan; 5 Department of Genome Biology, Faculty of Medicine, University of Tsukuba, Ibaraki, 305-8575 Japan; 6 JEM Utilization Center, Human Spaceflight Technology Directorate, JAXA, Ibaraki, 305-8505 Japan

**Keywords:** Spleen, Lymph node

## Abstract

Secondary lymphoid organs are critical for regulating acquired immune responses. The aim of this study was to characterize the impact of spaceflight on secondary lymphoid organs at the molecular level. We analysed the spleens and lymph nodes from mice flown aboard the International Space Station (ISS) in orbit for 35 days, as part of a Japan Aerospace Exploration Agency mission. During flight, half of the mice were exposed to 1 *g* by centrifuging in the ISS, to provide information regarding the effect of microgravity and 1* g* exposure during spaceflight. Whole-transcript cDNA sequencing (RNA-Seq) analysis of the spleen suggested that erythrocyte-related genes regulated by the transcription factor GATA1 were significantly down-regulated in ISS-flown vs. ground control mice. GATA1 and Tal1 (regulators of erythropoiesis) mRNA expression was consistently reduced by approximately half. These reductions were not completely alleviated by 1 *g* exposure in the ISS, suggesting that the combined effect of space environments aside from microgravity could down-regulate gene expression in the spleen. Additionally, plasma immunoglobulin concentrations were slightly altered in ISS-flown mice. Overall, our data suggest that spaceflight might disturb the homeostatic gene expression of the spleen through a combination of microgravity and other environmental changes.

## Introduction

During spaceflight, astronauts experience several hostile environments, such as altered gravity, high-dose space radiation, and the psychological stress from an isolated and confined space, which affect several physiological systems in the human body^1^. In particular, previous studies indicated that spaceflight has a profound impact on the immune system^2,3^. Clinical events potentially related to the immune system and the activation of latent viruses in astronauts have been reported^4,5^. Specimens taken from astronauts, mainly blood samples, have been used to monitor the influence of spaceflight on the immune system^6–16^. These studies suggested that spaceflight affects various immune parameters such as the distribution of leukocytes^6,11^, granulocyte and monocyte function^6,13^, natural killer cell function^14^, and cytokine levels in the plasma^8,15^ and in response to stimuli^11,16^. However, the molecular and cellular mechanisms underlying these effects of spaceflight remain elusive. Moreover, the impacts of spaceflight on immune organs and tissues within living individuals are usually difficult to investigate as human specimens can only be obtained by low-invasive approaches. To address these issues, several space-based experiments have been conducted using animal models^17^.

Secondary lymphoid organs such as lymph nodes, Peyer’s patches, and the spleen are integral to efficient immune response and provide microenvironments for cell-cell communication^18^. Previous studies have shown that spaceflight causes a reduction in the spleen mass of mice^19–22^ and rats^23,24^. A reduction of spleen mass was also observed in mice receiving hind limb unloading^25^, which is considered as a ground-based model experiment of the spaceflight environment. In addition, a recent study showed that socio-environmental stressors, which astronauts might be subject to during spaceflight, also caused a reduction of spleen mass in mice^26^.

Consistent with the overall mass change, the numbers of mouse spleen cells apparently also decrease during spaceflight^19,21,22^. However, results regarding changes in the cell sub-population in the spleen are inconsistent, most likely due to differences in mission duration, housing conditions, and genetic background^19,22,27–31^. As spaceflight causes several environmental changes^1^, it is difficult to determine the environmental change responsible for the physiological disturbance caused by spaceflight.

The Japan Aerospace Exploration Agency (JAXA) has recently established a new experimental platform known as the Multiple Artificial-gravity Research System (MARS)^32^. MARS monitors the effect of altered gravity on live mice, by centrifuging mouse cages to control the gravity experienced. JAXA conducted space experiments using MARS in the International Space Station (ISS) during orbit for 35 days, and then returned all the mice back to the earth in a live condition. In this mission, each male mouse inhabited an individual cage, thereby avoiding any unexpected fighting between mice that could possibly have an undesirable effect on the immune system. Notably, a group of mice was exposed to 1 *g* during spaceflight through the centrifugation of cages. This is expected to help elucidate the influence of microgravity in orbit and the effect of 1 *g* exposure during spaceflight.

In this study, we aimed to elucidate the impacts of spaceflight on the spleen and lymph nodes at the molecular level. We investigated gene expression and structure of the murine spleen and lymph nodes recovered from the JAXA mission (Mouse habitat Unit-1; MHU-1). We found that, in the spleen, but not in lymph nodes, spaceflight significantly down-regulated some genes required for erythrocyte function (e.g., *Hba-a1*), the expression of which is controlled by the transcription factor GATA binding protein 1 (GATA1).

## Results

### Effect of microgravity on spleen mass

We used MARS to analyse 12 male mice housed on board the ISS for 35 days^32^. Half of the launched mice (six mice) were exposed to microgravity in orbit (hereafter referred to as Micro-G or MG) for 35 days and the other six mice were exposed to 1 *g* by centrifuging in the ISS (hereafter referred to as Artificial 1G or AG); other environmental conditions caused by spaceflight were practically identical. After landing, samples of the spleen and lymph nodes were obtained.

**Figure 1:**
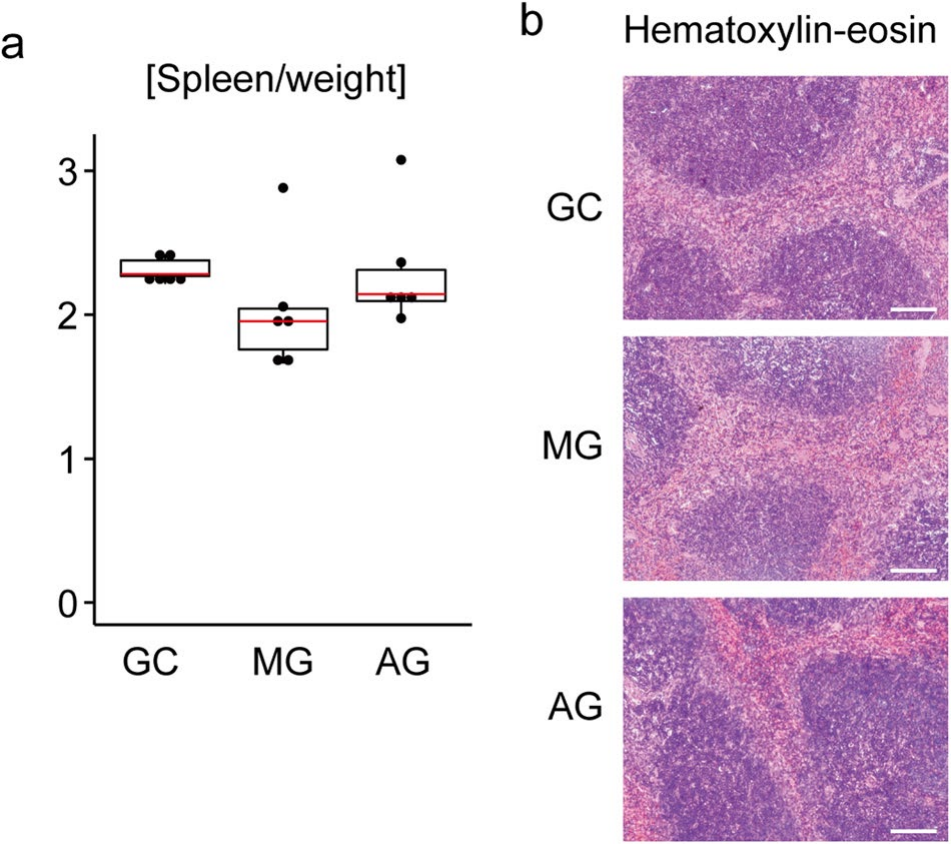
Effect of spaceflight on spleen mass and structure. (**a**) Ratio of spleen weight to total body weight in spaceflight and control mice. N = 6. Both box plots and dot plots are shown in the figure. The red line indicates medians. GC: ground control mice; MG: mice flown in the ISS; AG: mice receiving 1 *g* by centrifugation in the ISS. (**b**) Haematoxylin-eosin staining of paraffin-embedded spleen sections. Scale bars indicate 100 µm. Data represent a typical example of three independent mouse samples.

The ratio of spleen weight to body weight tended to be reduced in MG mice as compared with that of the ground control (GC) mice (Fig. 1a). Moreover, the exposure of 1 *g* by centrifugation was apparently able to recover the reduction caused by the environments of the ISS. However, the difference did not reach a significant level (GC vs MG, P = 0.054 by the Mann-Whitney U test; P = 0.165 by the Student’s t-test: MG vs AG, P = 0.09307 by the Mann-Whitney U test; P = 0.310 by the Student’s t-test) owing to one large outlier each in MG and AG (GC vs MG without outlier, P = 6.05 × 10^−3^ by the Mann-Whitney U test; P = 0.026 by the Student’s t-test: MG vs AG without outliers, P = 0.032 by the Mann-Whitney U test; P = 0.035 by the Student’s t-test). Thus, it is likely that the reduction of spleen mass after spaceflight may be partially due to MG.

Haematoxylin-eosin (HE) staining of paraffin-embedded spleen sections showed that mice flown in the ISS appear to have a normal splenic structure with white and red pulps (Fig. 1b), implying that spaceflight could cause a relatively non-selective reduction of spleen cells.

### Effect of spaceflight on gene expression in the spleen

**Figure 2:**
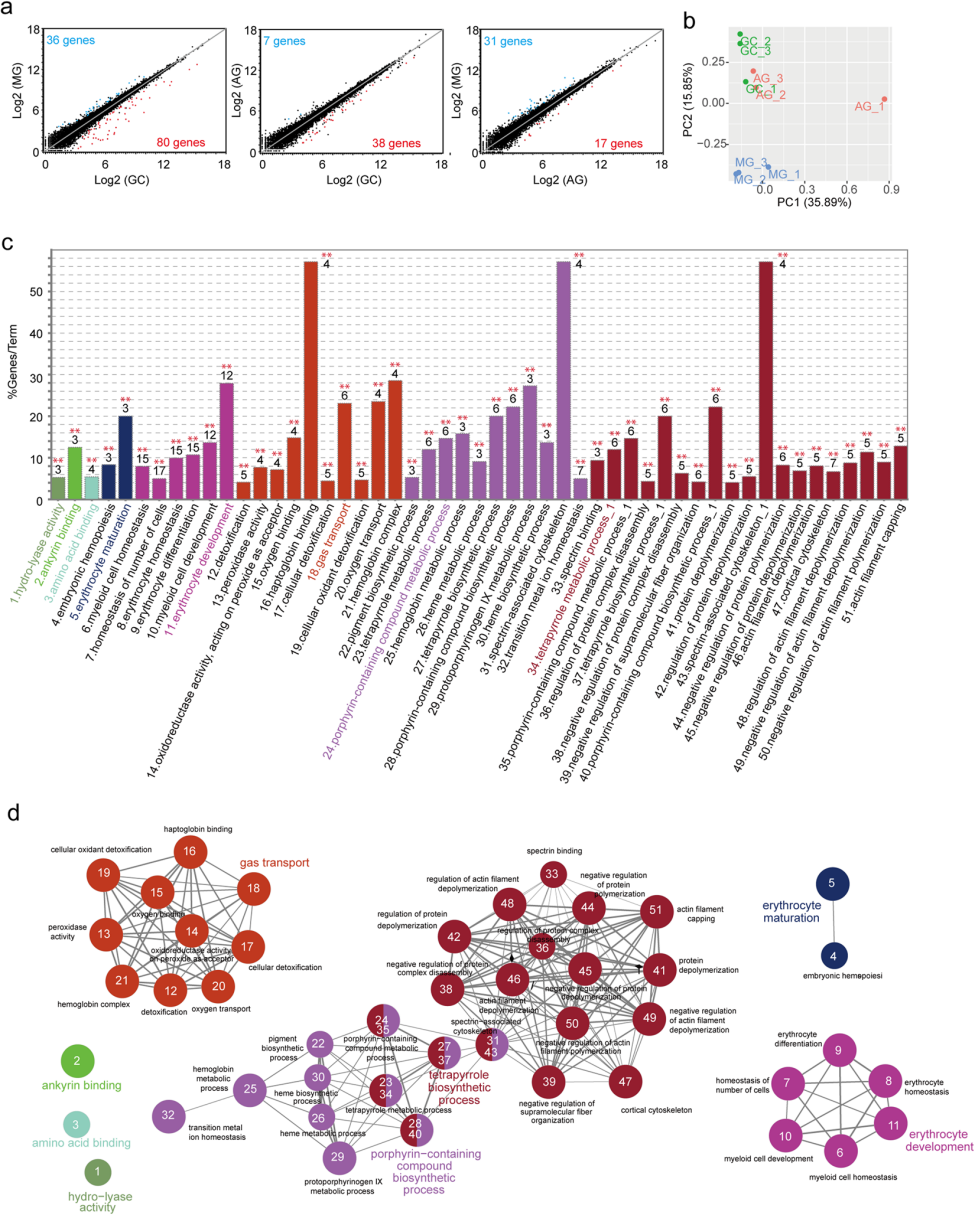
Down-regulation of erythrocyte-related genes in the spleen by spaceflight. (**a**) Scatter plots of RNA-Seq data. Each axis shows log2 of average gene expression values, which were normalized using the CLC Main Workbench (Qiagen). GC: ground control mice; MG: mice flown in the ISS; AG: mice receiving 1 G by centrifugation in the ISS. Significantly up- and down-regulated genes (FDR P-value < 0.05 and fold change >±2 estimated by the EDGE test) are indicated as red and blue dots, respectively. (**b**) PCA analysis of gene expression in the spleen of MG (blue), AG (red), and GC (green) mice. (**c**) Gene ontology (GO) enrichment analysis of genes down-regulated in the spleens of MG mice as compared to GC mice. GO terms and percentage of the down-regulated genes among total termed genes are exhibited. Top number of each bar indicates the number of reduced genes in each term. **P < 0.01, Two-sided hypergeometric test corrected with Bonferroni step down. (**d**) Clusters of GO terms of genes significantly down-regulated in the spleens of MG compared to GC mice. Each circle shows a GO term. A line between circles shows a correlation of two GO terms. A number in each GO term circle corresponds to number of the GO term in (**b**).

RNA-Seq analysis of gene expression in the spleens of MG, AG, and GC mice showed that the largest difference in gene expression profile was between MG and GC mice, suggesting a considerable impact of environmental change consequent to spaceflight (Fig. 2a and Supplementary Table S1). Principal component analysis (PCA) showed that the gene expression profiles of MG mice are considerably different from those of AG and GC mice whereas the gene expression profiles between AG and GC are relatively similar (close) except for one AG mouse (Fig. 2b). The results of PCA therefore suggest an impact of microgravity on gene expression of the spleen in mice.

Gene ontology (GO) enrichment analysis suggested a significant reduction of numerous GO terms in MG mice as compared to GC mice (Fig. 2c). Some GO terms were correlated with each other and formed clusters, represented by GO terms of ‘erythrocyte development’, ‘porphyrin and tetrapyrolle biosynthesis process’, ‘gas transport’, and ‘erythrocyte maturation’ (Fig. 2d) except for 3-independent GO terms, ‘ankyrin binding’, ‘amino acid binding’, and ‘hydro-lyase activity’. Because porphyrin, a tetrapyrolle compound, is a critical structure of the haeme essential for oxygen transport by erythrocytes, these terms might be practically equivalent to maturation, development, and function of erythrocytes. These results indicated that spaceflight causes a reduction of gene sets related to erythrocyte development and function. Notably, some genes related to erythrocyte development and function were also significantly reduced in AG as compared to GC mice and in MG compared to AG mice (Supplementary Figs S1, S2 and Supplementary Table S1) although substantively fewer genes exhibited reduced expression than the numbers obtained in the comparison between MG and GC conditions. Consequently, it is likely that the spaceflight environment as a combination of microgravity and other factors (e.g., radiation and stressors) impacts the expression of genes regulating erythrocyte development and function in the spleen.

**Figure 3:**
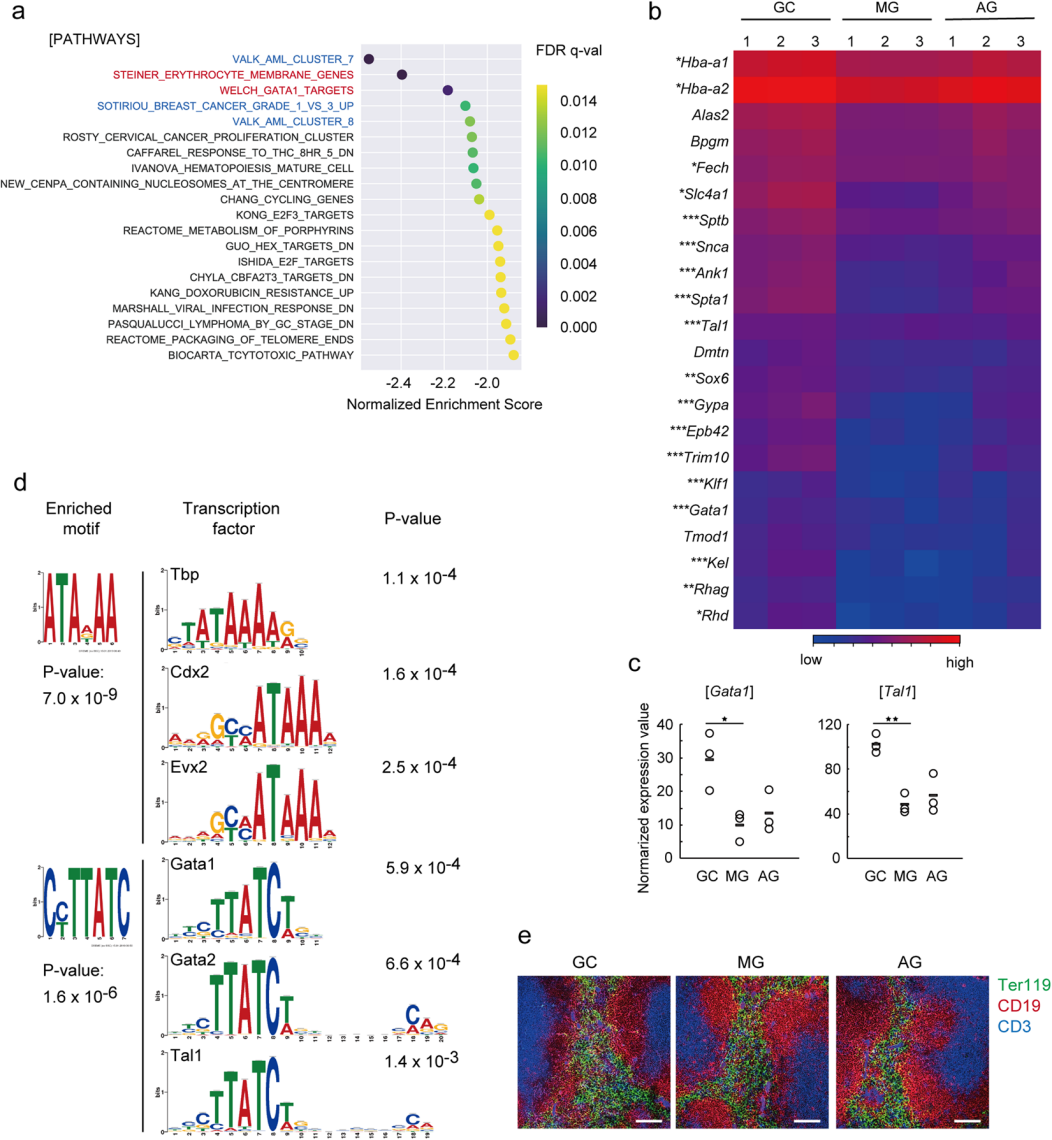
Down-regulation of GATA1 and Tal1-dependent genes in the spleen by spaceflight. (**a**) Gene set enrichment analysis (GSEA) of genes down-regulated in the spleens of MG compared to GC mice. Normalized enrichment score of each term is plotted. Dot colour indicates FDR q-value of enrichment. Erythrocyte-related pathway and GATA-1-related pathway are coloured red. Tumour-related pathways are coloured blue. (**b**) Heat map of representative gene expression in the spleens of GC, MG, and AG mice. Significantly up- and down-regulated genes are indicated as red and blue dots, respectively. *GATA1 target gene, **Tal1 target gene, ***GATA1 and Tal1 target gene as determined using ChIP-Atlas. (**c**) Normalized gene expression of *Gata1* and *Tal1* transcripts in the spleen. Bar indicates the average value. **P < 0.01, *P < 0.05, by EDGE test. (**d**) *In silico* prediction of transcription factors that bind to a region around to transcription start site (TSS) of genes down-regulated in the spleens of MG compared to GC mice. Enriched motifs (left) indicate genome sequence motifs significantly enriched in the TSS region (−200 bp to +100 bp) of the gene list down-regulated by MG compared with GC. The sequence motifs and P-values were determined using the DREAM algorithm. Subsequently, transcription factors that are predicted to bind to each enriched motif were determined using the TOMTOM algorithm. Names (upper left) and consensus binding sequence of transcription factors are indicated. P-values were determined by TOMTOM algorithm. (**e**) Immunostaining of spleen sections with TER119 (green), CD19 (red), and CD3e (blue). Scale bars indicate 100 µm. Data represent a typical example of three independent mouse samples.

We further performed gene set enrichment analysis (GSEA) on genes differentially expressed in the spleens of MG, AG, and GC mice. Notably, GSEA suggested a significant reduction of genes assigned to ‘transcription factor GATA1 target pathway’ and ‘erythrocyte membrane gene’ categories in the MG compared to GC, AG compared to GC, and MG compared to AG groups (Fig. 3a and Supplementary Figs S1 and S2). Moreover, in addition to GATA1-dependent genes, several genes controlled by the transcription factor T cell acute lymphocytic leukaemia 1 (Tal1) were down-regulated by spaceflight. As GATA1 and Tal1 promote the expression of genes controlling erythrocyte development^12,33^, the spaceflight-dependent reduction of genes related to erythroid development and functions might be due to an impairment of GATA1 and Tal1 activities. Consistent with this conjecture, several GATA1- and Tal1-depenent genes were down-regulated in the spleen by spaceflight (Fig. 3b). Moreover, the mRNA expression levels of GATA1 and Tal1 were significantly down-regulated in the spleens of MG and AG mice (Fig. 3c).

In order to further confirm the reduction in GATA1 and Tal1-dependent gene expression by spaceflight, we analysed plausible promoter regions of genes down-regulated by spaceflight. Data analysis using the DREAM algorithm^34^ revealed that two sequence motifs were significantly enriched around the transcription start site (TSS) region in genes commonly down-regulated in the spleens of MG and AG mice (Fig. 3d). Notably, subsequent analysis using the TOMTOM algorithm^35^ revealed that one of the enriched motifs shows significant similarity with binding sequences of GATA1, GATA2, and Tal1. Overall, these analyses of RNA-Seq data suggest that a decrease in GATA1 and Tal1 expression caused by spaceflight may be responsible for the reduction of several erythrocyte-related genes.

We then performed immunostaining of the spleen with erythrocyte marker TER-119, T cell marker CD3, and B cell marker CD19. The T cell zone and B cell zone were clearly detected in all samples. Although the expression level of erythrocyte-related genes was reduced in the spleen of MG and AG mice, the distribution of TER-119 positive cells did not change appreciably in these mice. Thus, the reduction of erythrocyte-related genes in the spleen did not largely influence the cell number and localization of erythrocytes.

### Effect of spaceflight on the amount of immunoglobulin in plasma

**Figure 4:**
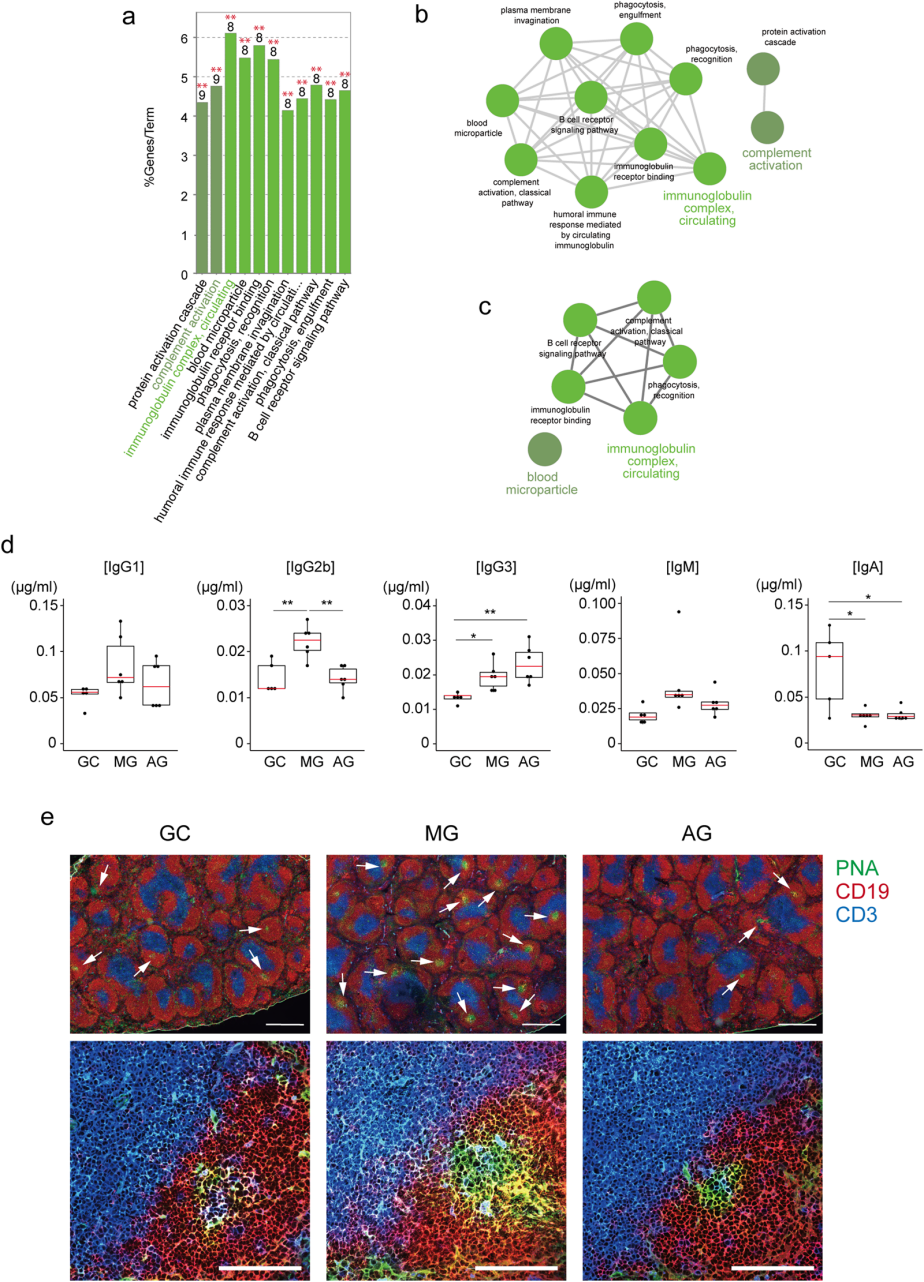
Disturbance of plasma immunoglobulin levels by spaceflight. (**a**) Gene ontology (GO) enrichment analysis of genes up-regulated in the spleens of MG compared to GC mice. GO terms and percentage of the down-regulated genes among total termed genes are shown. Top number of each bar indicates the number of reduced genes in each term. **P < 0.01, Two-side hypergeometric test corrected with Bonferroni step down. (**b**) Clusters of enriched GO terms for genes significantly up-regulated in the spleens of MG compared to GC mice. Each circle shows a GO term. A line between circles shows a correlation of two GO terms. (**c**) Clusters of enriched GO terms for genes significantly up-regulated in the spleens of AG compared to GC mice. Each circle shows a GO term. A line between circles shows a correlation of two GO terms. (**d**) Quantitative determination of immunoglobulin in sera from spaceflight and control mice as determined by ELISA. Both box plots and dot plots are shown in the figure. The red line indicates medians. GC: ground control mice; MG: mice flown in the ISS; AG: mice receiving 1 *g* by centrifugation in the ISS. **P < 0.01, *P < 0.05 by two-tailed Student’s t-test. (**e**) Immunohistochemical staining of spleen sections with PNA (green), anti-CD19 (red), and anti-CD3e (blue). Scale bars indicate 100 µm for lower panels and 500 µm for upper panels. Arrows in upper panels indicate clear and large germinal centres in B cell follicles. Data represent a typical example of three independent samples.

The GO analysis of splenic gene expression also suggested that spaceflight and microgravity might alter the expression of a gene set with terms of ‘Immunoglobulin complex circulating’ (Fig. 4a and Supplementary Fig. S3). We therefore checked the immunoglobulin level in the plasma of MG, AG, and GC mice. Enzyme-linked immunosorbent analysis (ELISA) showed an increase in the plasma concentration of IgG2b and IgG3 in MG mice, and a decrease of IgA. The concentration of IgM and IgG1 was not significantly affected. The increment of IgG2b in MG plasma was reverted by the 1 *g* exposure whereas that of IgG3 was not. The reduction of IgA by spaceflight was also observed in the plasma of AG mice. Immunostaining of the spleen with peanut agglutinin lectin (PNA), a maker for germinal centre B-cells, showed a slight increment of PNA-positive cells in the B cell follicles in the spleens of MG mice (Fig. 4e). This may correlate with the disturbance of plasma immunoglobulin profile induced by spaceflight.

### Effect of spaceflight on gene expression in lymph nodes

**Figure 5:**
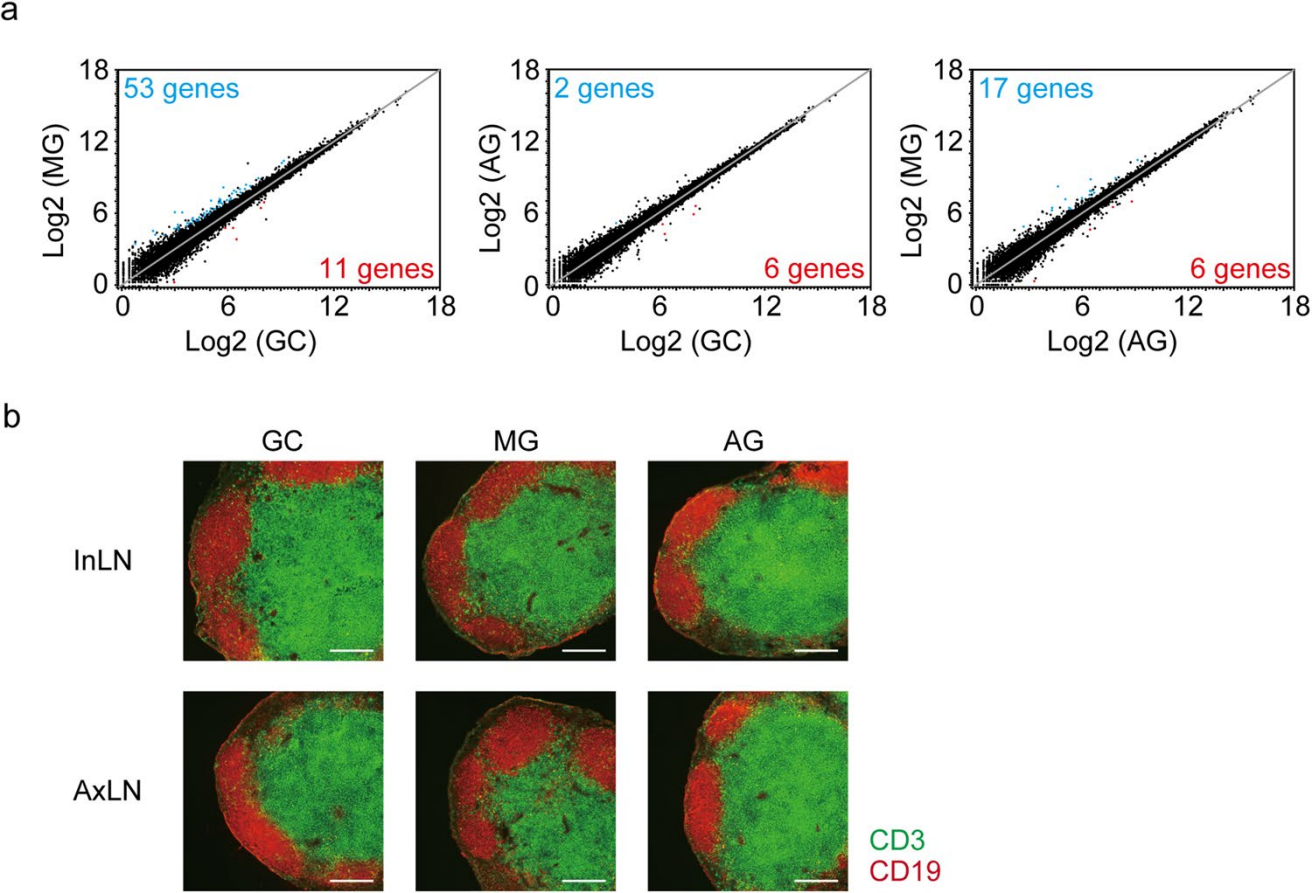
Minimal influences on gene expression and structure of lymph nodes by spaceflight. (**a**) Scatter plots of RNA-Seq data. Each axis shows log2 of average gene expression values, which were normalized using the CLC Main Workbench (Qiagen). GC: ground control mice; MG: mice flown in the ISS; AG: mice receiving 1 *g* by centrifugation in the ISS. Significantly up- and down-regulated genes (FDR p-value < 0.05 and fold change >±2 estimated using the EDGE test) are indicated as red and blue dots, respectively. (**b**) Immunostaining of inguinal (upper) and axillary (lower) lymph nodes with anti-CD3 (green) and anti-CD19 (red). Scale bars indicate 200 µm. Data represent a typical example of three independent mouse samples.

We also conducted RNA-Seq analysis of the inguinal lymph nodes of the spaceflight samples. Although some genes were significantly changed in the lymph nodes of MG mice as compared with GC and AG mice, the difference in expression level was subtle (Fig. 5a and Supplementary Table S2). Gene expression did not practically differ between the AG and GC samples (Fig. 5a). GO enrichment analysis showed no signature of enrichment in any altered genes. Moreover, enrichment sequence analysis did not show any significant consensus motifs in genes changed by spaceflight. Immunostaining showed no changes in B cell follicles or the T cell zone in lymph nodes (Fig. 5b). Overall, spaceflight may have a relatively minor impact on lymph node gene expression.

## Discussion

The 13-day Space Shuttle (STS) mission and the 30 day Bion M1 mission showed that the spleen mass of mice after spaceflight was reduced as compared with that of the ground control. Approximately 32% reduction in murine spleen mass was reported in the Bion M mission^21^. In contrast, a milder reduction was reported in the STS mission^20,27^. Consistent with these findings, our data on a 35 day mission in the ISS showed an average reduction of mass of approximately 12%, although the difference was not statistically significant. Notably, our MARS experiments revealed that the reduction of spleen mass is partially alleviated by centrifuging at 1 *g*, suggesting that long-term microgravity contributes to the reduction of spleen mass. One possible explanation is that bone loss consequent to microgravity indirectly influenced spleen mass. Accordingly, MG mice showed a significant reduction of bone loss as compared to that of AG mice. However, significantly reduced spleen mass in mice was only caused by short-^25,36^ but not longer term (3 weeks) hind limb unloading, whereas bone loss occurred with hind limb unloading under the latter condition^37^. Together, these studies implied that bone loss might not be sufficient for inducing a reduction of spleen mass. Therefore, the possibility that unidentified effects by microgravity may directly affect the size of the spleen cannot be ruled out.

In addition, other space environmental factors appear to be involved in the reduction of the spleen mass. Because the altitude of the Bion-M1 satellite’s on-orbit flight (at 550 km) was higher than that of conventional manned spaceflight (at about 400 km) such as the ISS and Space Shuttle, the radiation dose during flight appears to also be different^32,38^ (see also https://genelab-data.ndc.nasa.gov/). Thus, high dose radiation may contribute to the reduction of spleen mass reported in post-flight observations. In addition, a recent study revealed the influence of socio-environmental stress on spleen mass in mice^26^. Therefore, the effect of spaceflight on spleen size may be dependent on a combination of microgravity and changes in the space environment, such as high dose radiation and other socio-environmental stressors.

Our data further suggest that spaceflight causes a reduction in the expression level of genes related to erythrocytes in the spleen. Spaceflight reportedly caused a reduction of the red cell mass in astronauts^39^, which was proposed to be due to the suppression of erythropoiesis. In addition, a reduction in the number of erythroid cells in the spleen of rats after 22 day spaceflight was reported^23^. Notably, the results of colony formation assays suggest that erythropoiesis is reduced in the bone marrow of flight mice^40^. As extramedullary haematopoiesis occurs in the spleens of mice^41^, the mechanisms controlling the extramedullary erythropoiesis may be impaired in mice experiencing spaceflight.

RNA-Seq analysis and *in silico* promoter analysis suggested that the reduction of GATA1 and Tall-1 could be responsible for the down-regulation of the erythrocyte-related gene set (Fig. 2). The mechanism of reduction in the expression of these genes remains unclear. Notably, a previous study showed that simulated microgravity causes a reduction of *GATA1* mRNA in CD34^+^ haematopoietic stem and progenitor cells^42^. Moreover, macrophages derived from bone marrow in the space environment also showed reduced expression of GATA1 and GATA2^43^. Thus, microgravity may influence GATA-1 expression directly. Another possible explanation is that up-regulation of glucocorticoid induced by psychological stress may be responsible for the reduction in GATA-1 expression. Although glucocorticoid is not involved in erythropoiesis under steady state conditions^44,45^, it inhibits GATA-1 expression when stress erythropoiesis is induced by a severe decrement of erythrocytes consequent to anaemia and other conditions^46^. Because a down-regulation in GATA-1-dependent gene expression was observed upon comparison between MG and GC, and also between MG and AG, a combined effect of spaceflight environments likely caused the reduction of GATA-1- and Tal-1-dependent gene expression.

GO analysis also indicated that a change occurred in genes with the terms ‘hydro-lyase activity’, ‘ankyrin-binding’, and ‘amino acid binding’. Some of the genes (e.g., *Tal1*, *Alas2*) in these GO terms appear to be involved in erythrocyte development and function. However, it should be clarified in future studies how other genes along with these biochemical activities are correlated to space flight environments. Moreover, GSEA analysis showed that genes related to tumour-related pathways (‘Breast_Cancer_Grade_1_vs_3’, and ‘Acute myeloid leukemia_cluster’) were down-regulated by spaceflight. Building upon the results of a previous study showing a reduction of cancer-related genes after spaceflight^20^, future studies should investigate in further detail the possible connection between expression of tumour-related genes and spaceflight environments.

Previous studies using specimens of astronauts from a relatively short-term space flight (2 weeks) showed no significant changes in immunoglobulin levels in the plasma^6,47^. However, a long-term flight caused an increment in the level of total IgA and IgM in the serum^48^. Moreover, in the spleen of *Pleurodeles waltl*, the expression level of IgY heavy-chain was increased and the distribution of IgM VH genes was disturbed by spaceflight after a long-term flight^49,50^. In comparison, our data showed increments in the plasma levels of IgG2b and IgG3 and a reduction of IgA. Together, these findings support that the serum levels of immunoglobulins might be altered after a relatively long-term flight.

Consistent with this, GO analysis showed an up-regulation of genes with the term ‘immunoglobulin complex, circulating’, which is ascribed to the increase of immunoglobulin gene expression. This change in immunoglobulin gene expression may explain in part the disturbance in immunoglobulin plasma levels. Furthermore, the increment of IgG2b level in the plasma of mice from the MG condition was reverted by the 1 *g* exposure in the ISS. Expression of Ighg2b in the spleen of MG mice also increased as compared with that in GC and AG mice (Supplementary Table S1). This suggests that the long-term exposure of mice to microgravity might affect the mRNA expression of Ighg2 in the spleen, thereby changing the IgG2b protein level in the plasma. Conversely, specimens taken from astronauts showed no significant changes in the plasma levels of immunoglobulin after spaceflight^6,47,48^. However, our data showed no significant change in IgG1, which is most abundant in plasma; thus, further verification of the IgG subtype levels of astronaut blood should be performed.

Furthermore, our data showed that spaceflight causes a minimum change of gene expression in lymph nodes as compared to that in the spleen. This might arise because the spleen, but not lymph nodes, contains a high number of erythrocytes and has the ability of extramedullary haematopoiesis. However, it should be noted that the impact on expression of other genes unrelated to erythrocytes is also different between the spleen and lymph nodes, supporting the idea that the space environment might affect the properties of lymphocytes in a tissue-specific manner. Previous studies showed that the response of lymphocytes to mitogens differed between lymph nodes and the spleen in mice after spaceflight^51,52^. Future analysis in other secondary lymphoid organs such as tonsils and Peyer’s patches would be necessary to elucidate potential tissue specificity for the effect of space environments.

It should be noted that both MG and AG mice experienced hypergravity and stress during launching and landing. Thus, the impact of these events on gene expression should be considered. Moreover, a previous study showed a reduction in the splenic mass of mice receiving hypergravity via a centrifuge^53^. Therefore, it is important to elucidate the impact of hypergravity on gene expression in the spleen. In addition, it is inevitable that AG mice experienced microgravity during transfers from the launch rocket to the ISS and back^32^. Differences between AG and GC mice may therefore be due to the effect of short-term microgravity during these transfer processes. However, given that a short-term space flight afforded milder effects on spleen mass^20,27^, the influence of very short exposure of microgravity may not cause large impact on the spleen. This idea is supported by our data from PCA analysis (Fig. 2b), which suggested the relatively close profiles of gene expression between AG and GC rather than MG conditions. In addition to these concerns, a transportation period from landing to dissection of mice may also affect the data^32^. Notably, previous studies suggested that the effect of spaceflight on the spleen size and lymphocyte activity is relatively persistent and lasts for several days after the spaceflight^31^. This suggests that only a small effect might be derived from the transportation period on the spleen phenotype, which is consistent with our PCA analysis of gene expression profiles (Fig. 2b). However, in future studies, it would be important to prepare organ samples on board during the flight to determine the impact of microgravity by comparing between AG- and MG-conditioned mice.

Overall, our data suggest that relatively long-term spaceflight down-regulates the expression of genes related to erythrocytes in the spleen. This down-regulation is likely due to the reduction of transcription factors GATA-1 and Tal1, which control the expression of these genes. Detailed investigation of the possible association between the down-regulation of these gene and the development of anaemia during space flight should be addressed in future studies.

## Methods

### Mice

All mouse experiments were approved by the Institutional Animal Care and Use Committee of the University of Tsukuba, JAXA, Explore Biolabs, and NASA, and then conducted according to the applicable guidelines in Japan and the United States of America. Mice were maintained under specific pathogen-free conditions. The mice and treatment were described previously^32^. Briefly, the SpaceX Falcon 9 (SpX) rocket was launched on 18 July 2016 (EDT) from the Kennedy Space Center. After arriving at the ISS, the mice were housed there for 35 days. In the ISS, the mice were divided into two groups. Six mice were kept under microgravity and the other six were maintained under 1 *g* by centrifugation. The mice were then transferred to the SpX9 Dragon capsule, which later splashed down in the Pacific Ocean offshore from California on 26 August 2016 (GMT). Following splashdown, the mice were transported to the Long Beach Airport on 28 August 2016 (GMT), and then to a laboratory in San Diego (Explore Biolabs). The returned mice were euthanized and dissected at the laboratory to collect tissues.

### Immunostaining of tissue section

The spleen and lymph nodes were snap-frozen in OCT compound (Sakura Finetek Japan). Frozen tissues (6 μm thickness) were mounted on glass slides coated with amino silane. The spleen and lymph node sections were fixed with ice cold acetone for 5 min. After being blocked by 10% anti-goat serum in phosphate buffered saline (PBS), the spleen and lymph node sections were treated with primary antibodies in PBS containing 10% goat serum for 1 h at room temperature or overnight at 4 °C. After washing out the primary antibody solution with PBS, sections of the spleen and lymph nodes were further incubated with fluorescence-labelled secondary antibodies for 1 h. After washing out the secondary antibody solution, sections were covered with a glass coverslip using a mounting solution containing glycerol. Confocal colour images were obtained using a Leica confocal laser scanning microscope at 20× magnification. At least three different sections were analysed for each sample.

### RNA-Seq analysis

The RNA-Seq method was described previously in detail^54^. Briefly, total RNA was extracted from spleens and inguinal lymph nodes using TRIzol reagent according to the manufacturer’s protocol (Thermo Fisher Scientific, Waltham, MA). The RNA-Seq library was prepared from 50 ng of RNA by using the ENBNext Ultra Directional RNA Library Prep Kit (New England Biolabs (NEB), Ipswich, MA) after the depletion of rRNA (NEB NEBNext rRNA Depletion Kit). Paired-end sequencing (2 × 36 bases) was carried out by using NextSeq.500 (Illumina, San Diego, CA). Sequence reads were mapped to the mouse genome (mm10) using CLC Genomics Workbench (Version 7.5.1; Qiagen, Redwood City, CA). The expression level of each gene was estimated as ‘normalization values’ by CLC Genomics Workbench or CLC Main Workbench^55^. Differential expression was analysed by empirical analysis using the Empirical Analysis of DGE tool (edgeR test) in CLC Main Workbench.

### *In silico* binding motif analysis

To determine the consensus sequence motifs enriched in the TSS region of the differentially expressed gene set, the nucleotide sequences of TSS regions (200 bp upstream and 100 bp downstream from the TSS annotation) for the differentially expressed genes were obtained based on the RefSeq transcript database. These sequences were used as input for enriched motif search by the DREME algorithm^34^. The enriched sequence motifs discovered by DREME were further compared against known transcription factor binding motifs using the TOMTOM algorithm^34^. The sequence motifs obtained were further analysed using the TOMTOM algorithm^35^ to predict matching transcription factors.

### Gene set expression analysis

GSEA (http://software.broadinstitute.org/gsea) software was used to investigate pathway analysis. Expression datasets and phenotype files were created and imported onto GSEA software. The C2.all.v6.2.symbols dataset was downloaded using GSEA software. Then, the GSEA process was performed as default weighted enrichment statistics, with the number of permutations at 1000 and ‘excluding smaller sets’ set as 10. The cut-off levels were considered with false discovery rates (FDR) < 0.25.

### GO enrichment analysis

ClueGO (version 2.5.3) was used for GO analysis using Cytoscape (version 3.6.0) software. Both up-regulated and down-regulated DEGs were applied and visualized using biological process, molecular function, immune system process, and cellular component. The cut-off levels were considered with P-values < 0.05.

### Statistics

The Mann-Whitney U and Student’s t-test was used for determining P-value. For RNA-Seq data analysis, the Exact Test of Robinson and Smyth was used, and FDR-corrected P-value was used for testing statistics.

## Supplementary information

Supplementary data

Supplementary Table 1

Supplementary Table 2

## Data Availability

All data that support the findings of this study are available from the corresponding author upon reasonable request. RNA-Seq data are deposited in database of DDBJ (The DNA Databank of Japan, https://www.ddbj.nig.ac.jp/) (accession number DRA008121 and DRA008122).
