# Supplementary material for: Down-regulation of GATA1-dependent erythrocyte-related genes in the spleens of mice exposed to a space travel
Source: Sci Rep. 2019 May 21;9:7654. doi: 10.1038/s41598-019-44067-9 (PMC6529412; doi:10.1038/s41598-019-44067-9)
Supplement: Supplementary file 1 — Supplementary data [file 41598_2019_44067_MOESM1_ESM.pdf]

Down-regulation of GATA1-dependent erythrocyte-related genes in the spleens of mice exposed to a space travel

Kenta Horie[1,#], Hiroki Sasanuma[2,3,#], Takashi Kudo[3,4,#], Shin-ichiro Fujita[3,5], Maki Miyauchi[1], Takahisa Miyao[1], Takao Seki[1], Nobuko Akiyama[1], Yuki Takakura[1], Miki Shimbo[3,4], Hyojung Jeon[3,4], Masaki Shirakawa[3,6], Dai Shiba[3,6], Nobuaki Yoshida[2,3], Masafumi Muratani[3,5], Satoru Takahashi[3,4] & Taishin Akiyama[1,3,*]

[1]RIKEN Center for Integrative Medical Sciences, Yokohama 230-0045, Japan.

[2]Laboratory of Developmental Genetics, Institute of Medical Science, The University of Tokyo, Tokyo 108-8639, Japan.

[3]Mouse Epigenetics Project, ISS/Kibo experiment, Japan Aerospace Exploration Agency (JAXA), Ibaraki 305-8505, Japan.

[4]Laboratory Animal Resource Center and Department of Anatomy and Embryology, Faculty of Medicine, University of Tsukuba, Ibaraki 305-8575, Japan.

[5]Department of Genome Biology, Faculty of Medicine, University of Tsukuba, Ibaraki 305-8575, Japan.

[6]JEM Utilization Center, Human Spaceflight Technology Directorate, JAXA, Ibaraki 305-8505, Japan.

[#] These authors equally contributed to this work.

*Correspondence and requests for materials should be addressed to T.A. (email: taishin.akiyama@riken.jp)

Supplementary Fig. S1

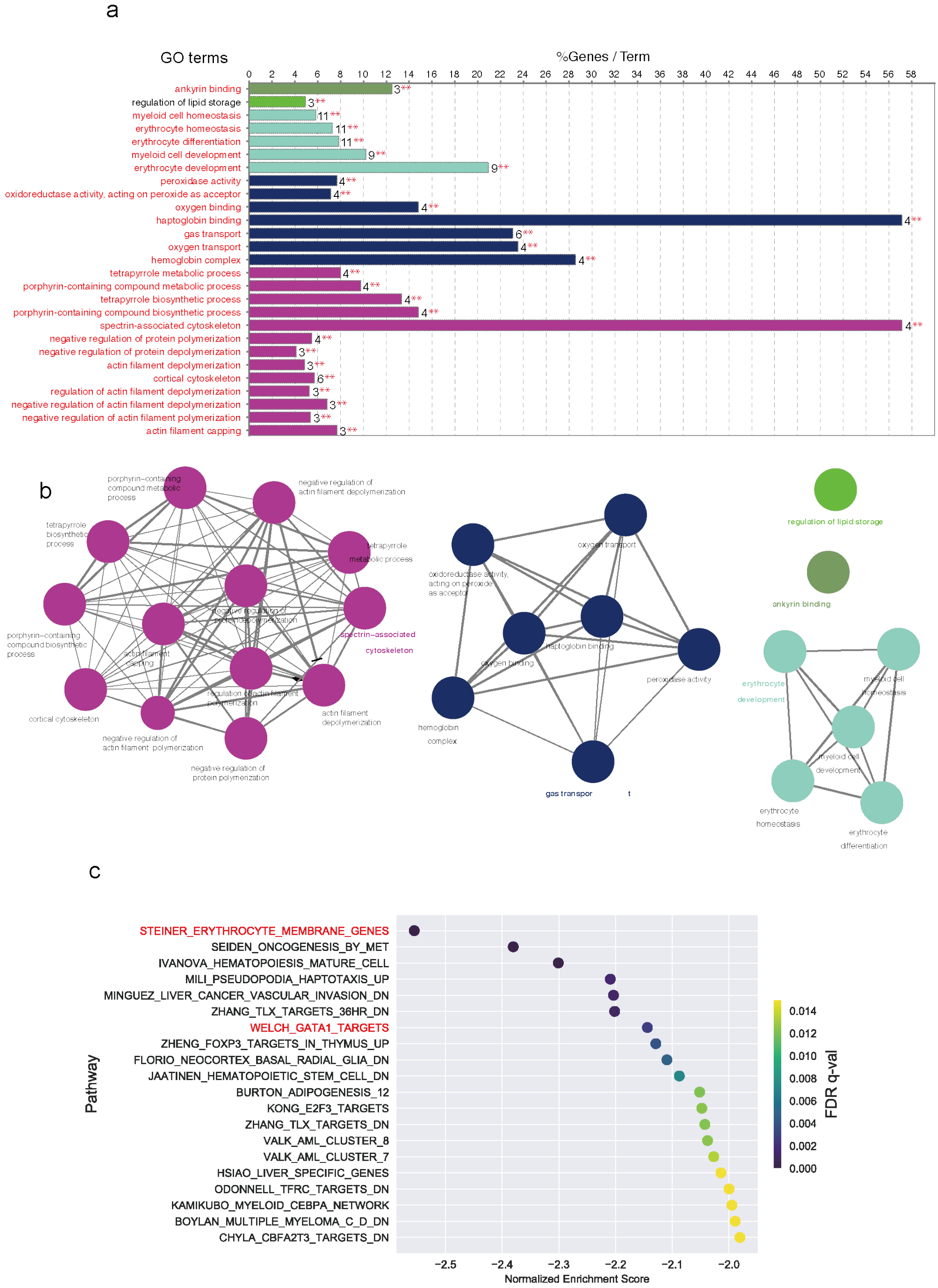

Supplementary Fig. S1.

a. Gene ontology enrichment analysis of genes down-regulated in the spleen of AG mice as compared to GC mice. GO terms and percentage of the down-regulated genes in total termed genes are exhibited. Top number of each bar indicates the number of reduced genes in each term. **$P < 0.01$, Two-side hypergeometric test corrected with Bonferroni step down. Red colored GO terms are common to that reduced in MG compared to GC (Fig. 2c).

b. Clusters of enriched GO terms for genes significantly down-regulated in the spleen of AG compared to GC. Each circle shows a GO term. A line between circles show the correlation of two GO terms.

c. Gene set enrichment analysis (GSEA) of genes down-regulated in the spleen of AG mice as compared to GC mice. Normalized enrichment score of each term are plotted. Dot color indicates FDR q-value of enrichment.

Supplementary Fig. S2

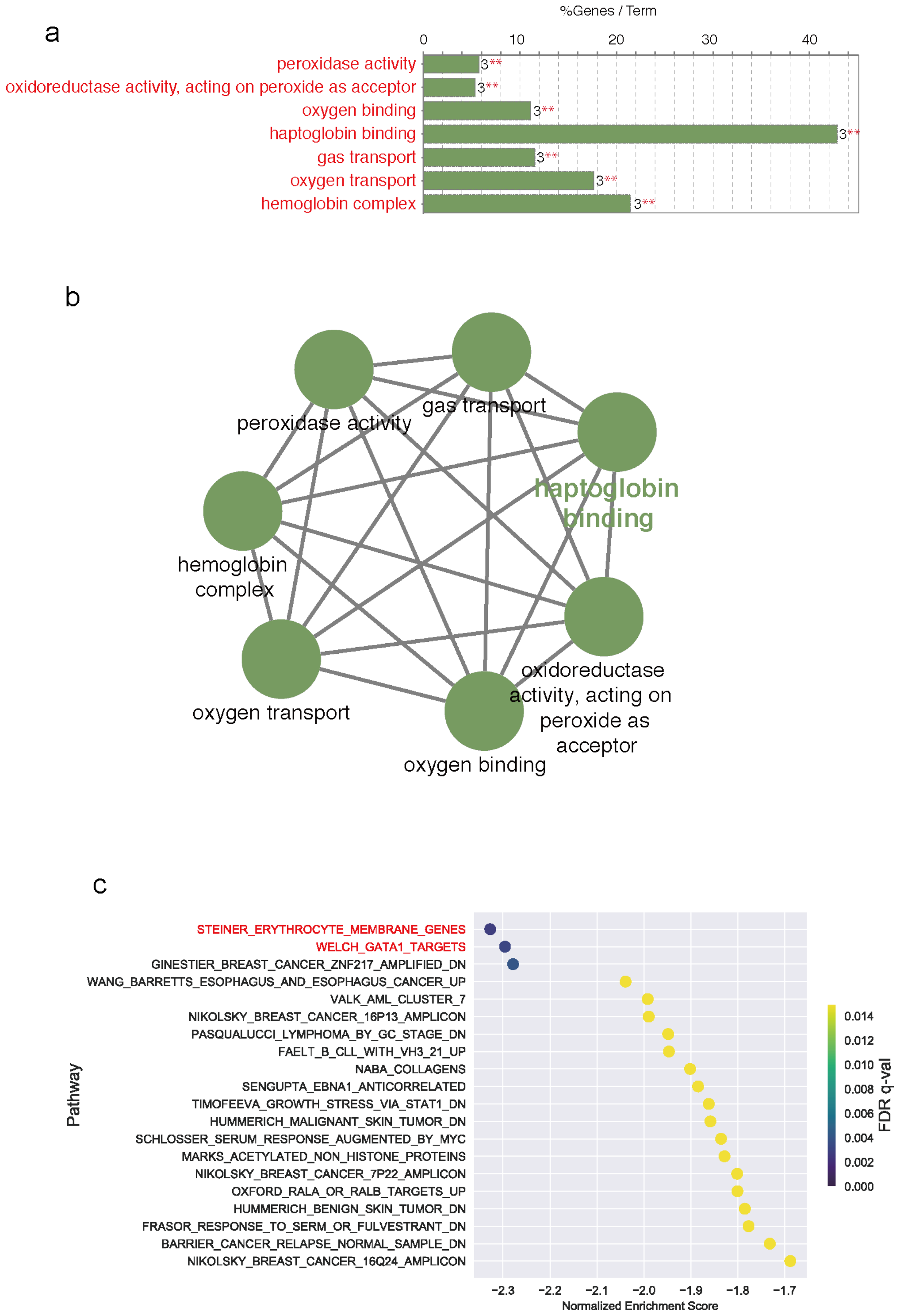

Supplementary Fig. S2.

a. Gene ontology enrichment analysis of genes down-regulated in the spleen of MG

mice as compared to AG mice. GO terms and percentage of the down-regulated genes in total termed genes are exhibited. Top number of each bar indicates the number of reduced genes in each term. **P < 0.01, Two-side hypergeometric test corrected with Bonferroni step down.

b. Clusters of enriched GO terms for genes significantly down-regulated in the spleen of MG compared to AG. Each circle shows a GO term. A line between circles show the correlation of two GO terms.

c. Gene set enrichment analysis (GSEA) of genes down-regulated in the spleen of MG mice as compared to AG mice. Normalized enrichment score of each term are plotted. Dot color indicates FDR q-value of enrichment.

Supplementary Fig. S3

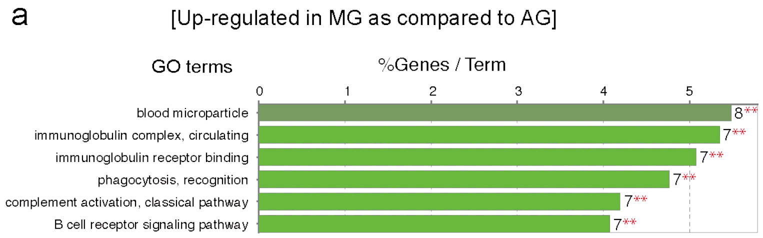

Supplementary Fig. S3

a. Gene ontology enrichment analysis of genes up-regulated in the spleen of MG mice compared to AG mice. GO terms and percentage of the down-regulated genes in total termed genes are exhibited. Top number of each bar indicates the number of reduced genes in each term. **P < 0.01, Two-side hypergeometric test corrected with Bonferroni step down.

Supplementary Table 1

Gene lists of differentially expressed genes in the spleen among MG, AG, and GC mice

Supplementary Table 2

Gene lists of differentially expressed genes in inguinal lymph nodes among MG, AG, and GC mice
